# Tobacco Product Use Among Adults — United States, 2017

**DOI:** 10.15585/mmwr.mm6744a2

**Published:** 2018-11-09

**Authors:** Teresa W. Wang, Kat Asman, Andrea S. Gentzke, Karen A. Cullen, Enver Holder-Hayes, Carolyn Reyes-Guzman, Ahmed Jamal, Linda Neff, Brian A. King

**Affiliations:** ^1^Office on Smoking and Health, National Center for Chronic Disease Prevention and Health Promotion, CDC; ^2^Center for Tobacco Products, Food and Drug Administration, Silver Spring, Maryland; ^3^Tobacco Control Research Branch, National Cancer Institute, National Institutes of Health, Bethesda, Maryland.

Cigarette smoking harms nearly every organ of the body and causes adverse health consequences, including heart disease, stroke, and multiple types of cancer (*1*). Although cigarette smoking among U.S. adults has declined considerably, tobacco products have evolved in recent years to include various combustible, noncombustible, and electronic products (*1*,*2*). To assess recent national estimates of tobacco product use among U.S. adults aged ≥18 years, CDC, the Food and Drug Administration (FDA), and the National Institutes of Health’s National Cancer Institute analyzed data from the 2017 National Health Interview Survey (NHIS). In 2017, an estimated 47.4 million U.S. adults (19.3%) currently used any tobacco product, including cigarettes (14.0%; 34.3 million); cigars, cigarillos, or filtered little cigars (3.8%; 9.3 million); electronic cigarettes (e-cigarettes) (2.8%; 6.9 million); smokeless tobacco (2.1%; 5.1 million); and pipes, water pipes, or hookahs (1.0%; 2.6 million). Among current tobacco product users, 86.7% (41.1 million) smoked combustible tobacco products, and 19.0% (9.0 million) used ≥2 tobacco products. By univariate analyses, the prevalence of current use of any tobacco product was higher among males than among females; adults aged <65 years than among those aged ≥65 years; non-Hispanic American Indian/Alaska Natives, whites, blacks, or multiracial adults than among Hispanics or non-Hispanic Asians; adults who lived in the South or Midwest than among those in the West or Northeast; adults who had a general educational development certificate (GED) than among those with other levels of education; adults who earned an annual household income of <$35,000 than among those with those with higher income; lesbian, gay, or bisexual adults than among heterosexual/straight adults; and adults who were divorced/separated/widowed or single/never married/not living with a partner than among those who were married/living with a partner. Prevalence was also higher among those who were uninsured, insured by Medicaid, or had some other public insurance than among those with private insurance or Medicare only; those who had a disability/limitation than among those who did not; and those who had serious psychological distress than among those who did not. Full implementation of evidence-based tobacco control interventions that address the diversity of tobacco products used by U.S. adults, in coordination with regulation of tobacco product manufacturing, marketing, and sales, can reduce tobacco-related disease and death in the United States (*1*–*3*).

NHIS is an annual, nationally representative, in-person survey of the noninstitutionalized U.S. civilian population (*4*). The 2017 Sample Adult component included 26,742 adults aged ≥18 years; the response rate was 53.0%. Data were weighted to adjust for differences in selection probability and nonresponse and to provide nationally representative estimates. Five tobacco products were assessed: cigarettes; cigars (cigars, cigarillos, or filtered little cigars); pipes (regular pipes, water pipes, or hookahs)*; e-cigarettes; and smokeless tobacco (chewing tobacco, snuff, dip, snus, or dissolvable tobacco). Current cigarette smokers were those who reported having smoked ≥100 cigarettes during their lifetime and smoked every day or some days at the time of survey. Current users of all other tobacco products were those who reported their use every day or some days at the time of survey. Prevalence estimates for current use of any tobacco product, any combustible tobacco product (cigarettes, cigars, or pipes), and use of ≥2 tobacco products^†^ were calculated. Estimates were calculated overall and separately by sex, age, race/ethnicity, U.S. region,^§^ education, marital status, annual household income, sexual orientation,^¶^ health insurance coverage,** disability,^††^ and presence of serious psychological distress.^§§^ T-tests were performed to assess overall differences in tobacco use between 2016 and 2017, with statistical significance defined as p<0.05.^¶¶^

Among U.S. adults in 2017, 19.3% (estimated 47.4 million) currently used any tobacco product and 16.7% (41.1 million; 86.7% of current tobacco users) used any combustible tobacco product (Table). Cigarettes were the most commonly used tobacco product (14.0%; 34.3 million), with the prevalence of cigarette smoking in 2017 being the lowest measured among U.S. adults since NHIS data collection for this measure began in 1965 (Figure 1). Prevalence estimates of other tobacco products in 2017 were as follows: cigars (3.8%; 9.3 million); e-cigarettes (2.8%; 6.9 million); smokeless tobacco (2.1%; 5.1 million); and pipes (1.0%; 2.6 million). During 2016–2017, declines occurred in current use of any tobacco product; any combustible tobacco product; ≥2 tobacco products; cigarettes; and smokeless tobacco (all p<0.05). Among current tobacco product users, the proportion who were daily users was 75.0% for cigarettes, 58.2% for smokeless tobacco, 40.5% for e-cigarettes, 12.4% for cigars, and 10.6% for pipes.

**TABLE:** Percentage of adults aged ≥18 years who reported tobacco product use "every day" or "some days," by tobacco product and selected characteristics — National Health Interview Survey, United States, 2017

Characteristic	Tobacco product use % (95% CI)							
	Any tobacco product*	Any combustible tobacco product^†^	Cigarettes^§^	Cigars/Cigarillos/Filtered little cigars^¶^	Regular pipe/Water pipe/Hookah**	E-cigarettes^††^	Smokeless tobacco^§§^	≥2 tobacco products^¶¶^
**Overall**	**19.3 (18.6–20.0)**	**16.7 (16.1–17.3)**	**14.0 (13.4–14.6)**	**3.8 (3.5–4.1)**	**1.0 (0.9–1.2)**	**2.8 (2.5–3.1)**	**2.1 (1.9–2.3)**	**3.7 (3.4–4.0)**
**Sex**								
Male	24.8 (23.8–25.8)	20.8 (19.9–21.7)	15.8 (15.0–16.7)	6.8 (6.2–7.4)	1.8 (1.5–2.1)	3.3 (2.8–3.7)	4.0 (3.6–4.5)	5.7 (5.1–6.2)
Female	14.2 (13.4–15.0)	12.9 (12.1–13.7)	12.2 (11.4–13.0)	1.0 (0.8–1.2)	0.4 (0.2–0.5)	2.4 (2.0–2.7)	0.2 (0.1–0.3)	1.8 (1.5–2.0)
**Age group (yrs)**								
18–24	18.3 (16.2–20.3)	14.0 (12.2–15.8)	10.4 (8.8–12.0)	4.3 (3.4–5.3)	2.5 (1.7–3.2)	5.2 (3.9–6.5)	2.9 (2.1–3.7)	5.2 (4.1–6.2)
25–44	22.5 (21.4–23.7)	19.5 (18.4–20.6)	16.1 (15.1–17.1)	4.7 (4.1–5.3)	1.2 (0.9–1.5)	3.6 (3.1–4.2)	2.5 (2.2–2.9)	4.7 (4.2–5.3)
45–64	21.3 (20.1–22.5)	18.9 (17.8–20.0)	16.5 (15.4–17.5)	3.9 (3.4–4.4)	0.6 (0.4–0.8)	2.4 (2.0–2.7)	2.0 (1.7–2.3)	3.5 (3.1–4.0)
≥65	11.0 (10.1–11.8)	9.8 (9.0–10.7)	8.2 (7.4–9.0)	1.8 (1.4–2.1)	0.7 (0.5–0.9)	0.7 (0.5–0.9)	0.9 (0.6–1.2)	1.1 (0.8–1.4)
Race/Ethnicity***								
White, non-Hispanic	21.4 (20.6–22.2)	18.3 (17.5–19.0)	15.2 (14.4–15.9)	4.0 (3.6–4.4)	1.1 (0.9–1.3)	3.3 (2.9–3.6)	2.8 (2.5–3.1)	4.2 (3.8–4.5)
Black, non-Hispanic	20.1 (18.3–21.9)	18.8 (17.0–20.5)	14.9 (13.1–16.6)	6.0 (4.8–7.2)	1.4 (0.7–2.0)	2.2 (1.5–2.9)	0.6 (0.3–1.0)	4.1 (3.0–5.1)
Asian, non-Hispanic	8.9 (7.1–10.8)	8.0 (6.2–9.8)	7.1 (5.5–8.8)	—^†††^	—	0.9 (0.4–1.4)	—	1.2 (0.5–1.8)
American Indian/ Alaska Native, non-Hispanic	29.8 (18.9–40.7)	26.3 (16.5–36.0)	24.0 (14.4–33.5)	5.8 (3.2–8.3)	—	—	—	4.9 (2.3–7.5)
Hispanic	12.7 (11.4–14.0)	11.2 (9.9–12.4)	9.9 (8.6–11.1)	2.2 (1.5–2.8)	0.6 (0.3–0.8)	1.8 (1.1–2.5)	0.7 (0.4–1.0)	1.9 (1.3–2.6)
Multirace, non-Hispanic	27.4 (22.4–32.3)	23.8 (19.0–28.6)	20.6 (16.0–25.2)	4.3 (2.2–6.4)	—	5.6 (2.7–8.5)	—	6.4 (3.3–9.4)
**U.S. Census region^§§§^**								
Northeast	15.6 (13.8–17.4)	13.9 (12.3–15.6)	11.2 (9.8–12.6)	3.2 (2.5–3.8)	0.6 (0.3–0.9)	2.0 (1.5–2.6)	1.3 (0.9–1.8)	2.5 (1.8–3.1)
Midwest	23.5 (22.1–24.8)	20.5 (19.2–21.7)	16.9 (15.5–18.2)	4.9 (4.2–5.6)	1.4 (1.0–1.7)	2.9 (2.4–3.4)	2.9 (2.5–3.4)	4.7 (4.0–5.3)
South	20.8 (19.6–22.0)	18.0 (16.9–19.2)	15.5 (14.4–16.7)	4.1 (3.6–4.7)	0.9 (0.7–1.2)	3.1 (2.6–3.6)	2.2 (1.8–2.5)	4.1 (3.5–4.6)
West	15.9 (14.6–17.1)	13.4 (12.4–14.3)	11.0 (10.1–11.8)	2.8 (2.3–3.3)	1.2 (0.9–1.6)	2.8 (2.2–3.3)	1.7 (1.2–2.1)	3.0 (2.5–3.5)
**Education (adults aged ≥25 yrs)**								
0–12 yrs (no diploma)	26.1 (24.0–28.3)	24.1 (22.0–26.2)	23.1 (21.0– 25.2)	3.6 (2.5–4.7)	—	2.1 (1.5–2.8)	1.8 (1.2–2.4)	4.3 (3.1–5.4)
GED	42.6 (38.2–46.9)	38.5 (34.3–42.8)	36.8 (32.7–41.0)	6.4 (4.1–8.7)	—	7.2 (4.8–9.6)	3.4 (1.8–4.9)	9.9 (7.1–12.7)
High school diploma	24.3 (22.8–25.8)	21.2 (19.7–22.6)	18.7 (17.4–20.1)	4.1 (3.3–4.8)	0.7 (0.4–1.0)	3.1 (2.5–3.7)	2.8 (2.3–3.4)	4.4 (3.7–5.2)
Some college, no degree	23.1 (21.6–24.6)	19.6 (18.1–21.0)	17.4 (16.0–18.7)	3.4 (2.6–4.1)	1.0 (0.6–1.3)	3.4 (2.7–4.0)	2.3 (1.8–2.8)	3.8 (3.1–4.6)
Associate degree (academic or technical/vocational)	20.4 (18.6–22.2)	18.2 (16.5–19.9)	15.5 (13.9–17.1)	3.6 (2.9–4.4)	0.8 (0.4–1.2)	2.7 (2.0–3.4)	1.9 (1.4–2.5)	3.6 (2.8–4.4)
Undergraduate degree (bachelor’s)	12.5 (11.3–13.6)	10.7 (9.6–11.7)	7.1 (6.2– 7.9)	3.8 (3.2–4.5)	1.0 (0.6–1.3)	1.7 (1.2–2.2)	1.5 (1.1–1.8)	2.3 (1.8–2.8)
Graduate degree (Master's, doctoral or professional	8.3 (7.0–9.5)	7.5 (6.3–8.7)	4.1 (3.3–5.0)	3.2 (2.4–4.0)	0.9 (0.6–1.3)	0.9 (0.5–1.2)	0.8 (0.5–1.1)	1.4 (0.9–1.9)
**Marital status**								
Married/Living with partner	17.6 (16.7–18.4)	15.0 (14.3–15.8)	12.4 (11.6–13.1)	3.6 (3.2–4.0)	0.7 (0.6–0.9)	2.3 (2.0–2.6)	2.1 (1.8–2.4)	3.1 (2.7–3.5)
Divorced/Separated/Widowed	23.1 (21.8–24.4)	21.1 (19.8–22.3)	19.1 (17.8–20.3)	3.4 (2.8–4.0)	0.7 (0.5–0.9)	2.9 (2.4–3.3)	1.7 (1.3–2.0)	4.0 (3.4–4.5)
Single/Never married/Not living with partner	21.0 (19.7–22.4)	17.9 (16.7–19.2)	14.4 (13.2–15.6)	4.6 (3.9–5.2)	2.1 (1.6–2.6)	4.1 (3.3–4.9)	2.2 (1.8–2.7)	5.0 (4.3–5.7)
**Annual household income ($)^¶¶¶^**								
<35,000	26.0 (24.6–27.3)	23.7 (22.4–25.1)	21.4 (20.1–22.7)	4.4 (3.7–5.1)	1.4 (1.1–1.7)	3.6 (3.1–4.1)	1.6 (1.3–1.9)	5.2 (4.5–5.9)
35,000–74,999	20.5 (19.4–21.6)	17.7 (16.7–18.8)	15.3 (14.3–16.3)	3.6 (3.1–4.2)	1.0 (0.7–1.3)	3.1 (2.6–3.6)	2.6 (2.1–3.0)	4.3 (3.7–4.9)
75,000–99,999	18.4 (16.6–20.1)	14.9 (13.3–16.6)	11.8 (10.3–13.4)	3.7 (2.7–4.7)	0.8 (0.4–1.1)	2.5 (1.7–3.2)	2.8 (2.1–3.4)	2.9 (2.1–3.7)
≥100,000	13.5 (12.3–14.7)	11.2 (10.1–12.2)	7.6 (6.7–8.4)	4.0 (3.4–4.6)	0.8 (0.5–1.1)	1.8 (1.3–2.2)	2.0 (1.6–2.4)	2.3 (1.9–2.8)
**Sexual orientation**								
Heterosexual/Straight	19.0 (18.3–19.8)	16.5 (15.9–17.1)	13.7 (13.1–14.4)	3.8 (3.5–4.1)	1.0 (0.8–1.2)	2.6 (2.4–2.9)	2.1 (1.9–2.3)	3.6 (3.2–3.9)
Lesbian/Gay/Bisexual	27.3 (23.0–31.6)	23.4 (19.4–27.4)	20.3 (16.7–24.0)	3.8 (2.2–5.5)	2.1 (0.9–3.2)	7.5 (5.3–9.8)	—	6.6 (4.8–8.5)
Health insurance coverage****								
Private insurance	16.2 (15.5–16.9)	13.6 (12.9–14.3)	10.5 (9.9–11.1)	3.6 (3.2–3.9)	0.9 (0.7–1.1)	2.3 (2.0–2.6)	2.2 (2.0–2.5)	2.9 (2.5–3.2)
Medicaid	28.2 (26.0–30.4)	25.9 (23.7–28.0)	24.5 (22.4–26.6)	3.6 (2.7–4.5)	1.0 (0.6–1.4)	4.8 (3.7–5.9)	1.0 (0.7–1.4)	5.7 (4.6–6.8)
Medicare only (aged ≥65 yrs)	11.0 (9.5–12.5)	9.9 (8.5–11.3)	8.7 (7.3–10.1)	1.8 (1.1–2.4)	—	0.7 (0.4–1.1)	0.8 (0.4–1.1)	1.3 (0.8–1.9)
Other public insurance	26.8 (24.2–29.5)	23.2 (20.6–25.7)	20.4 (18.0–22.9)	5.7 (4.3–7.0)	1.4 (0.7–2.1)	3.1 (2.1–4.1)	3.4 (2.3–4.5)	5.1 (3.8–6.3)
Uninsured	31.0 (28.7–33.4)	27.8 (25.6–30.1)	24.7 (22.5–26.9)	6.0 (4.6–7.5)	1.9 (1.2–2.7)	4.6 (3.6–5.6)	2.6 (1.9–3.2)	7.3 (5.8–8.7)
**Disability/Limitation^††††^**								
Yes	25.0 (23.3–26.7)	22.4 (20.8–24.1)	20.7 (19.1–22.3)	3.4 (2.6–4.1)	1.1 (0.7–1.5)	3.3 (2.6–4.1)	2.1 (1.5–2.6)	4.5 (3.7–5.3)
No	18.8 (17.9–19.8)	16.1 (15.2–16.9)	13.3 (12.5–14.0)	3.7 (3.3–4.1)	1.1 (0.9–1.3)	2.7 (2.4–3.1)	2.1 (1.8–2.5)	3.4 (3.0–3.8)
**Serious psychological distress^§§§§^**								
Yes	40.8 (36.9–44.7)	36.4 (32.6–40.3)	35.2 (31.4–39.0)	4.4 (2.9–6.0)	—	7.9 (5.8–10.1)	—	7.3 (5.4–9.3)
No	18.5 (17.8–19.2)	16.0 (15.4–16.6)	13.2 (12.5–13.8)	3.8 (3.5–4.1)	1.1 (0.9–1.2)	2.6 (2.3–2.9)	2.1 (1.9–2.3)	3.5 (3.2–3.8)

**Abbreviations:** CI = confidence interval; E-cigarettes = electronic cigarettes; GED = general educational development certificate; HS = high school.

* Any tobacco product use was defined as use either every day or some days of at least one tobacco product. For cigarettes only, users were defined as persons who had smoked ≥100 cigarettes during their lifetime and now smoked cigarettes either every day or some days.

^†^ Any combustible tobacco product use was defined as use either every day or some days of at least one combustible tobacco product: cigarettes; cigars, cigarillos, or filtered little cigars; pipes, water pipes, or hookahs. For cigarettes only, users were defined as persons who had smoked ≥100 cigarettes during their lifetime and now smoked cigarettes every day or some days.

^§^ Current cigarette smokers were defined as persons who reported smoking ≥100 cigarettes during their lifetime and now smoked cigarettes every day or some days.

^¶^ Reported smoking cigars, cigarillos, or little filtered cigars at least once during their lifetime and now smoked at least one of these products every day or some days.

** Reported smoking tobacco in a regular pipe, water pipe, or hookah at least once during their lifetime and now smoked at least one of these products every day or some days.

^††^ Reported using electronic cigarettes at least once during their lifetime and now used e-cigarettes every day or some days.

^§§^ Reported using chewing tobacco, snuff, dip, snus, or dissolvable tobacco at least once during their lifetime and now used at least one of these products every day or some days.

^¶¶^ Use was defined as use either every day or some days of at least two or more of the following tobacco products: cigarettes (≥100 cigarettes during lifetime); cigars, cigarillos, or filtered little cigars; pipes, water pipes, or hookahs; electronic cigarettes; or smokeless tobacco products. Among multiple tobacco product users, 84.1% used two products, 13.4% used three products, and 2.5% used four or more tobacco products

*** Hispanic persons could be of any race.

^†††^ Dashes indicate that prevalence estimates with a relative standard error ≥30% are not presented.

^§§§^ *Northeast:* Connecticut, Maine, Massachusetts, New Hampshire, New Jersey, New York, Pennsylvania, Rhode Island, and Vermont; *Midwest:* Illinois, Indiana, Iowa, Kansas, Michigan, Minnesota, Missouri, Nebraska, North Dakota, Ohio, South Dakota, and Wisconsin; *South:* Alabama, Arkansas, Delaware, District of Columbia, Florida, Georgia, Kentucky, Louisiana, Maryland, Mississippi, North Carolina, Oklahoma, South Carolina, Tennessee, Texas, Virginia, and West Virginia; *West:* Alaska, Arizona, California, Colorado, Hawaii, Idaho, Montana, Nevada, New Mexico, Oregon, Utah, Washington, and Wyoming.

^¶¶¶^ Based on observed income as obtained from combined family income bracketing questions.

**** *Private coverage:* includes adults who had any comprehensive private insurance plan (including health maintenance organizations and preferred provider organizations). *Medicaid:* for adults aged <65 years, includes adults who do not have private coverage, but who have Medicaid or other state-sponsored health plans including Children’s Health Insurance Program (CHIP); also includes adults aged ≥65 years who do not have any private coverage but have Medicare and Medicaid or other state-sponsored health plans including CHIP. *Medicare only:* includes adults aged ≥65 years who only have Medicare coverage. *Other coverage*: includes adults who do not have private insurance, Medicaid, or other public coverage, but who have any type of military coverage, coverage from other government programs, or Medicare. *Uninsured:* includes adults who have not indicated that they are covered at the time of the interview under private health insurance, Medicare, Medicaid, CHIP, a state-sponsored health plan, other government programs, or military coverage. Insurance coverage is ‘as of time of survey’.

^††††^ Disability status was defined on the basis of self-reported presence of selected limitations including vision, hearing, cognition, and movement. Limitations in performing activities of daily living were defined using the question “Does [person] have difficulty dressing or bathing?” Limitations in performing instrumental activities of daily living were defined on the basis of responses to the question “Because of a physical, mental, or emotional condition, does [person] have difficulty doing errands alone such as visiting a doctor’s office or shopping?” Any disability was defined as a “yes” response pertaining to at least one of the limitations listed (vision, hearing, cognition, movement, activities of daily living, or instrumental activities of daily living). A random sample of half of the respondents from the 2017 Person File was asked about limitations and weights from the Family Disability Questions File were applied.

^§§§§^ Based on the Kessler psychological distress scale, a series of six questions that ask about feelings of hopelessness, sadness, nervousness, restlessness, worthlessness, and feeling like everything is an effort in the past 30 days. Participants were asked to respond on a Likert Scale ranging from “None of the time” (score = 0) to “All of the time” (score = 4). Responses were summed over the six questions; persons with a score of ≥13 were coded as having serious psychological distress, and respondents with a score <13 were coded as not having serious psychological distress.

**FIGURE 1:**
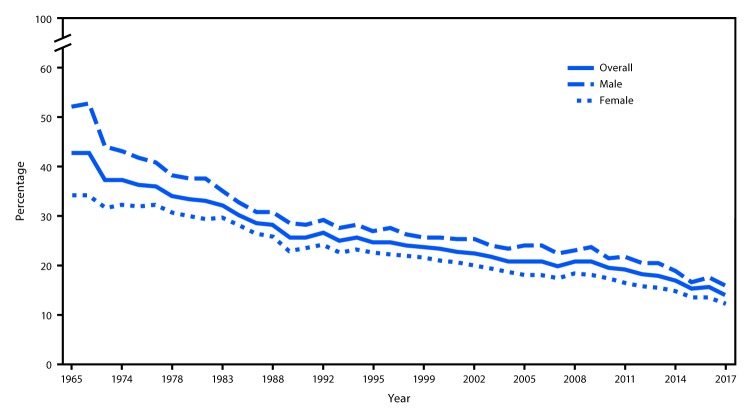
Percentage of adults aged ≥ 18 years who were current cigarette smokers,* overall and by sex — National Health Interview Survey (NHIS), United States, 1965–2017 * For NHIS years 1965–1991, current smokers included adults who reported that they had smoked ≥100 cigarettes in their lifetime and currently smoked. Since 1992, current smokers included adults who reported smoking ≥100 cigarettes during their lifetime and specified that they currently smoked every day or on some days. Data are not available for 1967–1969, 1971–1973, 1975, 1981, 1982, 1984, 1986, 1989, and 1996 because questions regarding smoking were not included in the NHIS conducted in those years. Related data and documentation can be found at https://www.cdc.gov/nchs/nhis/data-questionnaires-documentation.htm.

Overall, 3.7% of U.S. adults (9.0 million; 19.0% of current tobacco product users) used ≥2 tobacco products. Among multiple tobacco product users, 84.1% used two products, 13.4% used three products, and 2.5% used four or more products. The most prevalent tobacco product combinations were cigarettes and e-cigarettes (30.1%), followed by cigarettes and cigars (29.2%) (Figure 2).

**FIGURE 2:**
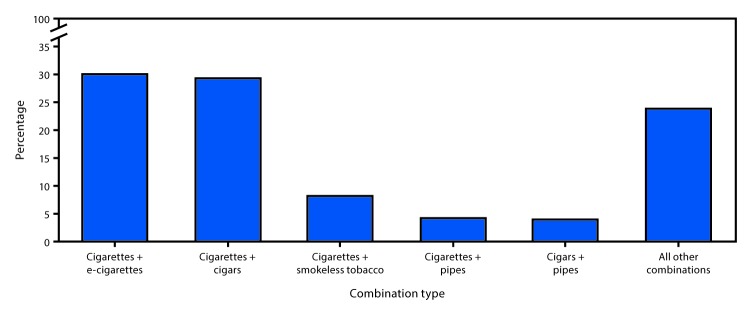
Top tobacco product use* combinations among adults aged ≥18 years who currently used ≥2 tobacco products^†^^,^^§^ — National Health Interview Survey, United States, 2017 * For cigarettes, current smokers were defined as persons who had smoked ≥100 cigarettes during their lifetime and now smoked either every day or some days. Current users of all other assessed tobacco products were defined as persons who reported use of each respective product every day or some days at the time of survey. ^†^ Percentages were calculated among adults who currently used ≥2 of the following five tobacco product types: cigarettes; cigars, cigarillos, or filtered little cigars (cigars); regular pipes, water pipe or hookahs (pipes); chewing tobacco, snuff, dip, snus, or dissolvable tobacco (smokeless tobacco); and electronic cigarettes (e-cigarettes). ^§^ A total of 26 distinct combinations were assessed (10 two-product type combinations; 10 three-product type combinations; 5 four-product type combinations, and 1 five-product type combination).

By univariate analyses, the prevalence of any current tobacco product use was higher among males (24.8%) than among females (14.2%); those aged 25–44 years (22.5%), 45–64 years (21.3%), or 18–24 years (18.3%) than among those aged ≥65 years (11.0%); non-Hispanic American Indian/Alaska Natives (29.8%), multiracial adults (27.4%), whites (21.4%), or blacks (20.1%) than among Hispanics (12.7%) or non-Hispanic Asians (8.9%); those who lived in the Midwest (23.5%) or the South (20.8%) than among those who lived in the West (15.9%) or Northeast (15.6%); those who had a GED (42.6%) than among those with other levels of education; those who were divorced/separated/widowed (23.1%) or single/never married/not living with a partner (21.0%) than among those married/living with a partner (17.6%); those who had annual household income of <$35,000 (26.0%) than among those with higher income; and lesbian, gay, or bisexual adults (27.3%) than among heterosexual/straight adults (19.0%). Prevalence was also higher among those who were uninsured (31.0%), insured by Medicaid (28.2%) or had some other public insurance (26.8%) than among those with private insurance (16.2%) or Medicare only (11.0%); those who had a disability/limitation (25.0%) than among those who did not (18.8%); and those who had serious psychological distress (40.8%) than among those who did not (18.5%).

## Discussion

Considerable progress has been made in reducing cigarette smoking among U.S. adults over the past half century: an estimated 14.0% of U.S. adults (34.3 million) were current cigarette smokers in 2017, representing a 67% decline since 1965. However, in 2017, nearly nine in 10 (41.1 million) adult tobacco product users reported using a combustible tobacco product, with cigarettes being the product most commonly used. The burden of death and disease from tobacco use in the United States is caused overwhelmingly by cigarettes and other combustible products, and an estimated 480,000 U.S. adults die from cigarette smoking and secondhand smoke exposure each year (*1*). Therefore, continued efforts to reduce all forms of combustible tobacco smoking, including cigarettes, among U.S. adults are especially important (*1*).

U.S. adults also report using various noncigarette tobacco products. In 2017, approximately one in five adults (47.4 million) currently used any tobacco product, and 19.0% of these adults reported multiple tobacco product use. Multiple tobacco product users are at increased risk for nicotine addiction and dependence (*1*,*5*). E-cigarettes were commonly used among multiple tobacco product users. Primary reasons for e-cigarette use among adults include curiosity, flavoring, cost, consideration of others, convenience, and simulation of cigarettes, as well as to attempt to quit smoking (*6*). However, although e-cigarettes could benefit adult smokers if used as a complete substitute for combustible tobacco smoking, evidence of the effectiveness of e-cigarettes as a cessation aid is inconclusive (*7*).

Demographic variations in tobacco product use were observed. For example, young adults reported the highest use of emerging products such as e-cigarettes and pipes; the higher prevalence of overall pipe use among young adults is likely primarily driven by water pipe or hookah use (*1*). Differences in tobacco product use across population groups might be related to multiple factors, including targeted advertising, differing perceptions regarding the relative harm or social acceptability of tobacco use, and differences in tobacco product prices and levels of access to cessation resources (*1*,*2*).

The findings in this report are subject to at least four limitations. First, the potential for recall bias exists because responses were self-reported and not biochemically validated. However, self-reported smoking status correlates highly with serum cotinine levels (*8*). Second, the questionnaire did not assess gender identity; including transgender persons could affect overall tobacco use estimates among the sexual and gender minorities considered in this report. Third, NHIS estimates are not generalizable to persons in the military or institutionalized populations. Finally, the NHIS Sample Adult component’s response rate of 53.0% might have resulted in nonresponse bias.

Full implementation of comprehensive tobacco control programs at the national, state, and local levels, including tobacco price increases, high-impact anti-tobacco mass media campaigns, comprehensive smoke-free laws,*** and barrier-free access to tobacco cessation counseling and approved medications, along with FDA regulation of tobacco products, can accelerate progress toward reducing tobacco-related death and disease in the United States (*3*). Given the increasing diversity of available tobacco products, coordinated efforts are key to implementing proven strategies while also exploring promising new strategies. For example, CDC supports the National Tobacco Control Program (*3*), and the Tips From Former Smokers campaign, which led to approximately half a million sustained quits among U.S. adult smokers during 2012–2015 (*9*). FDA launched the Every Try Counts campaign in 2018, which targets adults aged 25–54 years who have attempted to quit smoking in the last year but were unsuccessful. The campaign also complements FDA’s recently announced plan to explore reducing nicotine content in cigarettes to minimally or nonaddictive levels (*10*). The National Cancer Institute supports research to improve tobacco dependence treatment and provides resources to help smokers quit, including Smokefree.gov; the toll-free national quitline network (1-800-QUIT-NOW); and LiveHelp online. These coordinated strategies, in combination with state and local level tobacco prevention and control strategies that address the diversity of tobacco products, can reduce tobacco related disease and death in the United States (*1*).

**Summary**

**What is already known about this topic?**

Although cigarette smoking among U.S. adults has declined considerably, tobacco products have evolved in recent years to include various combustible, non-combustible, and electronic products.

**What is added by this report?**

In 2017, an estimated 47.4 million U.S. adults (19.3%) currently used any tobacco product. Among current tobacco product users, 86.7% (41.1 million) smoked combustible tobacco products, and 19.0% (9.0 million) used two or more tobacco products.

**What are the implications for public health practice?**

Implementation of evidence-based tobacco control interventions that address the diversity of tobacco products used by U.S. adults, in coordination with regulation of tobacco product manufacturing, marketing, and sales, can reduce tobacco-related disease and death in the United States.

## Footnotes

* The use of regular pipe, water pipe, or hookah was assessed together using a single question. Interviewers could read the following sentences if necessary: “A hookah is a type of water pipe. It is sometimes called a ‘narghile’ (NAR-ge-lee) pipe. Do not include electronic hookahs or e-hookahs”; “Do not include electronic pipes or e-pipes.”

† Current use of ≥2 tobacco products was defined as use either every day or some days of at least two or more of the following tobacco products: cigarettes (≥100 cigarettes during lifetime); cigars, cigarillos, or filtered little cigars; pipes, water pipes, or hookahs; electronic cigarettes; or smokeless tobacco products.

§ *Northeast:* Connecticut, Maine, Massachusetts, New Hampshire, New Jersey, New York, Pennsylvania, Rhode Island, and Vermont. *Midwest:* Illinois, Indiana, Iowa, Kansas, Michigan, Minnesota, Missouri, Nebraska, North Dakota, Ohio, South Dakota, and Wisconsin. *South:* Alabama, Arkansas, Delaware, District of Columbia, Florida, Georgia, Kentucky, Louisiana, Maryland, Mississippi, North Carolina, Oklahoma, South Carolina, Tennessee, Texas, Virginia, and West Virginia. *West:* Alaska, Arizona, California, Colorado, Hawaii, Idaho, Montana, Nevada, New Mexico, Oregon, Utah, Washington, and Wyoming.

¶ Sexual orientation was determined using the question “Which of the following best represents how you think of yourself?” Response options included “gay,” “straight, that is, not gay,” “bisexual,” “something else,” and “I don’t know the answer” among male respondents, and “lesbian or gay,” “straight, that is, not lesbian or gay,” “bisexual, “something else,” and “I don’t know the answer” among female respondents. Respondents were considered to be lesbian, gay, or bisexual if they responded “gay,” “lesbian or gay,” or “bisexual.”

** *Private coverage:* includes adults who had any comprehensive private insurance plan (including health maintenance organizations and preferred provider organizations). *Medicaid:* for adults aged <65 years, includes those who did not have private coverage, but who had Medicaid or other state-sponsored health plans, including Children’s Health Insurance Program (CHIP). For adults aged ≥65 years, includes adults aged ≥65 years who did not have any private coverage but had Medicare and Medicaid or other state-sponsored health plans including CHIP; *Medicare only:* includes adults aged ≥65 years who only had Medicare coverage; *Other coverage:* includes adults who did not have private insurance, Medicaid, or other public coverage, but who had any type of military coverage, coverage from other government programs, or Medicare. *Uninsured:* includes adults who did not indicate that they were covered at the time of the interview under private health insurance, Medicare, Medicaid, CHIP, a state-sponsored health plan, other government programs, or military coverage.

†† Disability status was defined on the basis of self-reported presence of selected limitations, including vision, hearing, cognition, and movement. Limitations in performing activities of daily living were defined using the question “Does [person] have difficulty dressing or bathing?” Limitations in performing instrumental activities of daily living were defined on the basis of responses to the question “Because of a physical, mental, or emotional condition, does [person] have difficulty doing errands alone such as visiting a doctor’s office or shopping?” Any disability was defined as a “yes” response pertaining to at least one of the limitations listed (vision, hearing, cognition, movement, activities of daily living, or instrumental activities of daily living). A random sample of half of the respondents from the 2017 Person File was asked about limitations, and weights from the Family Disability Questions File were applied.

§§ The Kessler psychological distress scale is a series of six questions that ask about feelings of hopelessness, sadness, nervousness, restlessness, worthlessness, and feeling like everything is an effort in the past 30 days. Participants were asked to respond on a Likert Scale ranging from “None of the time” (score = 0) to “All of the time” (score = 4). Responses were summed over the six questions; persons with a score of ≥13 were coded as having serious psychological distress, and respondents with a score <13 were coded as not having serious psychological distress.

¶¶ NHIS 2016 data were incorporated to inform statistically significant differences during 2016–2017 for the use of any tobacco product, any combustible tobacco product, ≥2 tobacco products, cigarettes, cigars, pipes, e-cigarettes, and smokeless tobacco. The 2016 Sample Adult component included 26,742 adults aged ≥18 years; the response rate was 54.3%.

*** Includes policies that completely prohibit smoking in all indoor areas of private-sector worksites, restaurants, and bars. https://www.cdc.gov/mmwr/volumes/65/wr/mm6524a4.htm.
